# Haplotype-Based Noninvasive Prenatal Diagnosis of 21 Families With Duchenne Muscular Dystrophy: Real-World Clinical Data in China

**DOI:** 10.3389/fgene.2021.791856

**Published:** 2021-12-14

**Authors:** Lingrong Kong, Shaojun Li, Zhenhua Zhao, Jun Feng, Guangquan Chen, Lina Liu, Weiqin Tang, Suqing Li, Feifei Li, Xiujuan Han, Di Wu, Haichuan Zhang, Luming Sun, Xiangdong Kong

**Affiliations:** ^1^ Department of Fetal Medicine & Prenatal Diagnosis Center, Shanghai First Maternity and Infant Hospital, School of Medicine, Tongji University, Shanghai, China; ^2^ Genetic and Prenatal Diagnosis Center, Department of Obstetrics and Gynecology, The First Affiliated Hospital of Zhengzhou University, Zhengzhou, China; ^3^ Celula (China) Medical Technology Co., Ltd., Chengdu, China

**Keywords:** cell-free DNA, duchenne muscular dystrophy, noninvasive prenatal diagnosis, haplotype, Bayes factor

## Abstract

Noninvasive prenatal diagnosis (NIPD) of single-gene disorders has recently become the focus of clinical laboratories. However, reports on the clinical application of NIPD of Duchenne muscular dystrophy (DMD) are limited. This study aimed to evaluate the detection performance of haplotype-based NIPD of DMD in a real clinical environment. Twenty-one DMD families at 7–12 weeks of gestation were prospectively recruited. DNA libraries of cell-free DNA from the pregnant and genomic DNA from family members were captured using a custom assay for the enrichment of *DMD* gene exons and spanning single-nucleotide polymorphisms, followed by next-generation sequencing. Parental haplotype phasing was based on family linkage analysis, and fetal genotyping was inferred using the Bayes factor through target maternal plasma sequencing. Finally, the entire experimental process was promoted in the local clinical laboratory. We recruited 13 complete families, 6 families without paternal samples, and 2 families without probands in which daughter samples were collected. Two different maternal haplotypes were constructed based on family members in all 21 pedigrees at as early as 7 gestational weeks. Among the included families, the fetal genotypes of 20 families were identified at the first blood collection, and a second blood collection was performed for another family due to low fetal concentration. The NIPD result of each family was reported within 1 week. The fetal fraction in maternal cfDNA ranged from 1.87 to 11.68%. In addition, recombination events were assessed in two fetuses. All NIPD results were concordant with the findings of invasive prenatal diagnosis (chorionic villus sampling or amniocentesis). Exon capture and haplotype-based NIPD of DMD are regularly used for DMD genetic diagnosis, carrier screening, and noninvasive prenatal diagnosis in the clinic. Our method, haplotype-based early screening for DMD fetal genotyping *via* cfDNA sequencing, has high feasibility and accuracy, a short turnaround time, and is inexpensive in a real clinical environment.

## Introduction

Duchenne muscular dystrophy (DMD, OMIM# 310200), the most common X-linked recessive inherited muscle disease, affects approximately 1 in 3,600–6,000 live male births (Walter and Reilich, 2017; Coote et al., 2018; Fox et al., 2020). DMD is usually not recognized by ultrasound examination or serum screening, and is often diagnosed after delivery. No gold standard treatment for DMD has been established to date (van Deutekom and van Ommen, 2003; Verhaart and Aartsma-Rus, 2019). Consequently, prenatal diagnosis is necessary for most DMD families. Traditional prenatal diagnosis is associated with an invasive procedure similar to chorionic villus sampling (CVS) or amniocentesis. However, this invasive procedure may lead to miscarriage or stillbirth (incidence: 0.1–1.3%), and is not applicable to patients with sampling contraindications (Agarwal and Alfirevic, 2012; Bakker et al., 2017; Salomon et al., 2019; Di Mascio et al., 2020).

The discovery of cell-free fetal DNA (cffDNA) in maternal circulation led to a new era of noninvasive prenatal testing (Lo et al., 1997; Chiu et al., 2002). Recently, several teams have reported using haplotype-based noninvasive prenatal diagnosis (NIPD) as an alternative solution to overcome these limitations while maintaining high accuracy (Xu et al., 2015; Yoo et al., 2015; Parks et al., 2016). Nevertheless, because of high costs or long turnaround times, cffDNA technologies for DMD remain at the experimental laboratory stage. The practicability of clinical applications of haplotype-based NIPD thus requires further evaluation. In clinical practice, the ideal platform for DMD prenatal diagnosis needs to be designed for fetal genotyping and be equally applicable to the proband and carrier. Previously, before detection of fetal genotype, multiplex ligation-dependent probe amplification plus Sanger sequencing required multiple steps to validate the mother as a carrier (Lalic et al., 2005). In this study, we developed a high-accuracy assay that can simultaneously be utilized for the NIPD of the fetal genotype and gene detection for the proband and mother.

In this study, we performed carrier detection and NIPD on 21 enrolled DMD families as a routine test. The procedure of sample collection, DNA extraction, variant calling, and haplotype analysis were all completed by the in-house staff of First Affiliated Hospital of Zhengzhou University, Zhengzhou, Henan, China.

## Materials and Methods

### Detection Workflow

**FIGURE 1:**
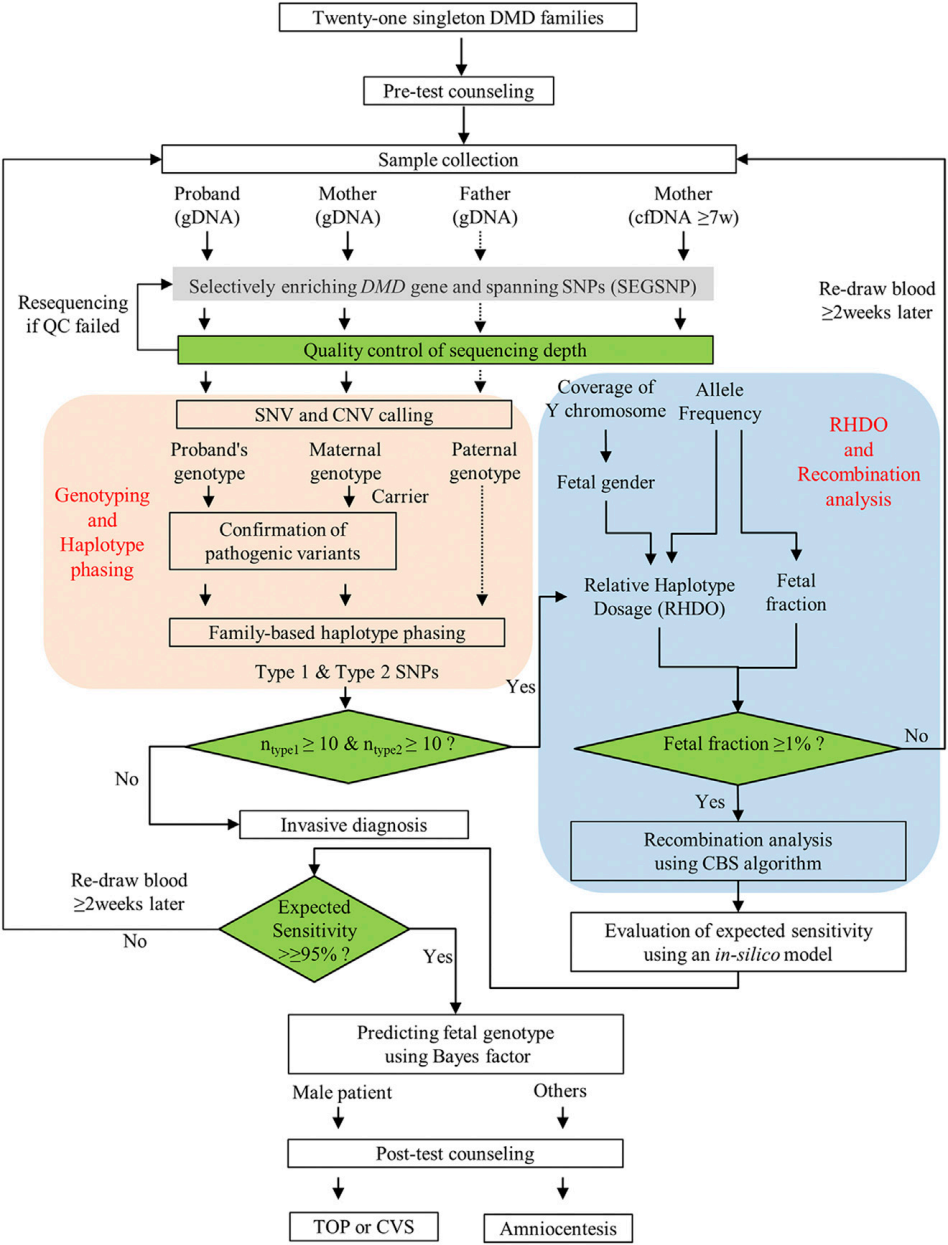
Workflow of NIPD of DMD. After genetic counseling, samples from the qualified 21 DMD family members were collected for non-invasive prenatal diagnosis (NIPD), and the results were released within 1 week when all samples met quality control requirements. Finally, a proper invasive diagnosis was recommended to confirm the accuracy of NIPD testing.

As shown in Figure 1, the SNP-based NIPT workflow involves several steps. First, whole blood samples from members of at-risk DMD families were collected. Then, the average depth of each sample was evaluated. The maternal pathogenic and wild-type haplotype were further constructed on the basis of family-based linkage analysis to ascertain type 1 SNPs and type 2 SNPs. Meanwhile, the cfDNA was also sequenced to calculate fetal gender, fetal fraction, and relative haplotype dosage (RHDO). Finally, combined with the results of haplotype phasing, the RHDO was further used in recombination analysis by the circular binary segmentation (CBS) algorithm and predicting fetal genotypes using the Bayes factor.

### Sample Information

Twenty-one DMD families (named P1-P21) were enrolled from December 2020 to August 2021 after genetic counseling and a receipt of informed consent (Supplementary Figure S1). The average gestational age of the recruited families was 10^+3^ weeks (Table 1). Fifteen families had complete pedigrees, whereas two families only included one daughter (P12 was unaffected, P18 was a carrier). Six families had incomplete pedigrees; i.e., no paternal samples were available (P1, P9, P13, P17, P19, and P21) (Supplementary Table S1). For each family, we collected 10 ml of peripheral blood from the pregnant mother and 2 ml of blood from the proband and father (when available). The study was approved by the Ethics Committee of First Affiliated Hospital of Zhengzhou University.

**TABLE 1:** Summary RHDO and prenatal diagnosis of the 21 families.

Family	Gestational weeks (w)	Pathogenic variants	RHDO						Invasive diagnosis
			FF^a^ (%)	Gender^b^	Type 1^c^	Type 2^d^	BF^e^	Result	
P1	11^+0^	EX53_55del	4.92	Male	354	354	2.70 × 10^47^	Affected	Affected
P2	12^+0^	EX46_48del	5.91	Female	311	180	1.80 × 10^80^	Carrier	Carrier
P3	11^+0^	EX12_13dup	5.50	Female	183	217	5.10 × 10^–29^	Normal	Normal
P4	12^+3^	c.3055C > T	6.21	Female	134	220	3.30 × 10^–28^	Normal	Normal
P5	9^+0^	EX45_50del	3.96	Male	365	365	1.40 × 10^–99^	Normal	Normal
P6	9^+5^	c.3786+2T > A	6.27	Male	528	528	2.70 × 10^162^	Affected	Affected
P7	8^+6^	EX10_11dup	8.21	Female	184	205	2.40 × 10^–110^	Normal	Normal
P8	10^+5^	EX3_4del	5.71	Female	198	166	5.00 × 10^38^	Carrier	Carrier
P9	9^+1^	EX8_26del	11.07	Female	459	459	6.90 × 10^–300^	Normal	Normal
P10	8^+0^	EX45_51del	1.87	Female	154	141	1.30 × 10^8^	Carrier	Carrier
P11	7^+3^	EX10_13del	5.54	Female	178	179	1.10 × 10^29^	Carrier	Carrier
P12	11^+5^	EX48_50del	7.14	Male	333	333	4.40 × 10^279^	Affected	Affected
P13	8^+0^	EX8_9dup	11.68	Female	274	274	1.10 × 10^180^	Carrier	Carrier
P14	9^+1^	EX50del	5.26	Female	120	108	4.90 × 10^9^	Carrier	Carrier
P15	11^+0^	EX45_55del	5.88	Male	406	406	6.30 × 10^–142^	Normal	Normal
P16	8^+0^	EX46_51del	7.58	Male	687	687	1.20 × 10^168^	Affected	Affected
P17	8^+1^	EX3_25dup	9.24	Male	349	349	1.00 × 10^–300^	Normal	Normal
P18	11^+5^	EX48_52del	6.37	Female	447	302	1.80 × 10^123^	Carrier	Carrier
P19	18^+0^	EX8_9dup	6.44	Female	131	131	3.20 × 10^31^	Carrier	Carrier
P20	13^+0^	EX45_47del	5.58	Female	97	152	4.40 × 10^22^	Carrier	Carrier
P21	13^+4^	EX8_9dup	6.32	Male	619	619	5.20 × 10^–173^	Normal	Normal

a Fetal fraction.

b Fetal gender.

c The number of informative alleles of the maternal pathogenic haplotype (Hap1).

d The number of informative alleles of the maternal wild-type haplotype (Hap2).

e Bayes factor.

### Probe Design for NIPD of DMD

**FIGURE 2:**
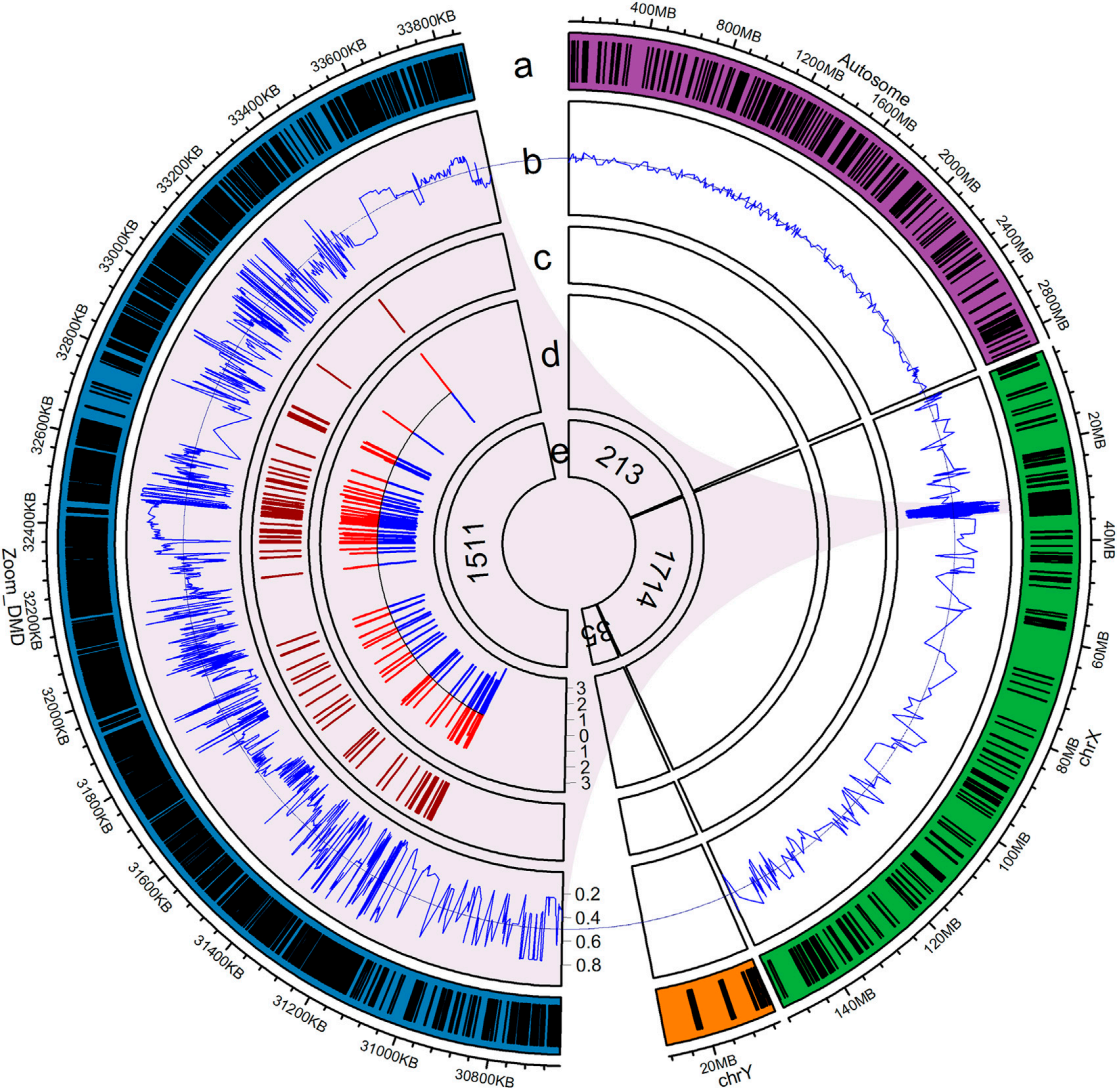
Illustration of the capture probe design. **(a)** Distribution of capture probes, with blue denoting the *DMD* gene that spans a 0.5-Mb region, green indicating the X chromosome, orange representing the Y chromosome, purple showing chromosomes 1–22, and dashes representing regions covered by capture probes; **(b)** Reference allele frequency of the SNPs in the East Asian population; **(c)** Position of exons of the *DMD* gene; **(d)** Intronic region adjacent to exons covered by capture probes, in log10 scale; **(e)** Number of SNPs in each region.

A 288.612-kb capture panel TargetSeq^®^ One kit (iGeneTech, China), enrichment of *DMD* gene exons and spanning SNPs, was designed to selectively enrich target regions based on the reference genome (GRCh37/hg19) (Figure 2). The probes covered all exonic regions (including untranslated regions), about 500-bp of intronic regions adjacent to exons, and 10,000-bp of flanking regions of the *DMD* gene. In addition, 1,511 common SNPs (MAF >0.10, 1000 Genomes Project Phase 3) spanning a 0.5-Mb region upstream and downstream of the *DMD* gene were included. To determine fetal gender and maternal chimerism, the probes also covered 35 sites on the Y chromosome and 203 common SNPs (MAF >0.45, 1000 Genomes Project Phase 3) on the X chromosome. Another 213 common SNPs (MAF >0.45, 1000 Genomes Project Phase 3) scattered on autosomes were used to calculate fetal fraction.

### Library Preparation and Next-Generation Sequencing

Circulating cell-free DNA (cfDNA) was extracted using a cfDNA extraction kit following the manufacturer’s instructions (Nahai Bio, Chengdu, China). Genomic DNA (gDNA) was extracted from the leucocytes of peripheral blood samples of the proband and parent (when the father was available) using an in-house protocol, and then fragmented into an average length of 200 bp. cfDNA and fragmented gDNA were subsequently captured after end-repair, barcode adapter ligation, and PCR amplification. The post-capture libraries were then subjected to PCR amplification and sequenced on the Ion Proton platform (Thermo Fisher Scientific, Lithuania).

For sequencing quality control, we calculated total reads, the average sequencing depth of target regions, areas with over 30× coverage, and on-target rates. In addition, second-time sequencing was required for both gDNA samples with an average sequencing depth of <30× and cfDNA samples with an average sequencing depth of <70×.

### Genotyping and Molecular Diagnosis of DMD

To detect DMD sequence variations in the mothers and probands, sequencing reads of gDNA were aligned to the human reference genome (GRCh37/hg19) using TMAP software (version 5.2.25). Small variants were identified with Torrent Variant Caller software (version 5.2.25) using default parameters, after removing duplicated reads. To identify microdeletions and duplications, we used the CNVkit (version 0.9.6) software (Talevich et al., 2016) with a sliding window of 200 bp and a step length of 100 bp using normal samples as reference. Variant annotations were accomplished by ANNOVAR (Wang et al., 2010). Finally, the genotypes of all family members were identified, and probands were confirmed to inherit the same pathogenic variants from the mother.

### Measurement of Fetal Fraction and Gender

We selected autosomal SNP loci as described in the probe design to calculate fetal fractions. After filtering out loci with depths of <100× or Phred quality scores of <13, we calculated the fetal fraction in maternal plasma (*f*) using the following equation: $f=\frac{a}{a+b}$ . We used the homologous locus in parents but with a different genotype, where *a* is the read depth of the fetal inherited paternal allele, and *b* is the read depth of the allele shared by the fetus and mother. The minimum requirement for fetal fraction was 1%, and samples below this threshold were re-sampled 2 weeks later.

Fetal gender was determined by the ratio between the average depth of Y loci and 213 autosomal SNP loci (chrY_ratio) in the maternal cfDNA. The threshold was set to 0.3%. A ratio of <0.3% indicated a female fetus; otherwise, it indicated a male fetus.

### Fetal Genotyping *via* Haplotype Analysis

**FIGURE 3:**
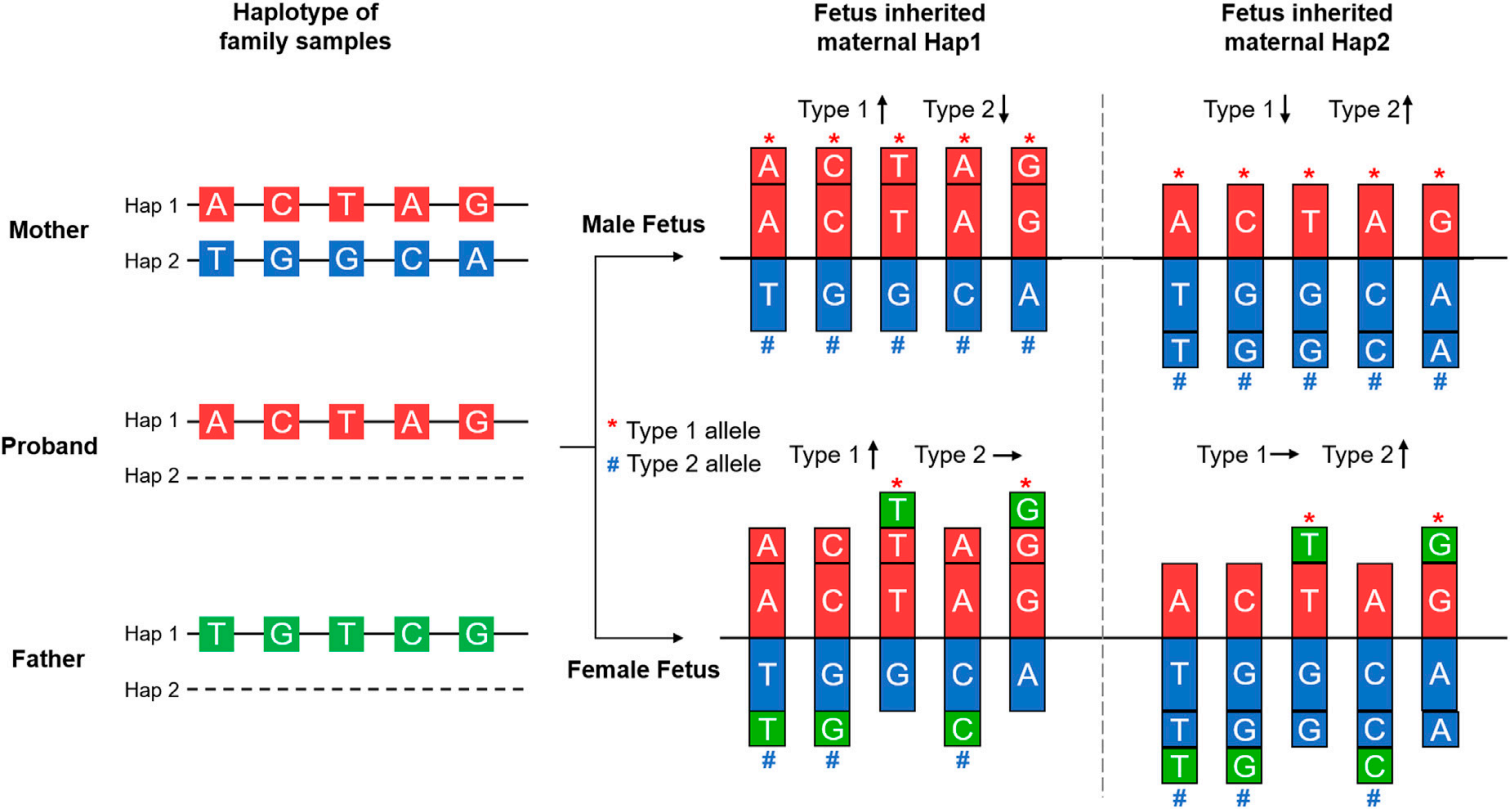
Principle of haplotype-based noninvasive prenatal diagnosis. Haplotype phasing of the SNPs in the *DMD* gene and its spanning region was achieved by Mendelian Law, as shown in the left part of the figure. The maternal haplotype was shared with the proband and carried the pathogenic variant; this was defined as Hap 1. The other haplotype was defined as Hap 2. For a male fetus, Type 1 and Type 2 alleles were the same as Hap 1 and Hap 2 alleles, respectively. For a female fetus, the paternal haplotype was also assessed. Only the Hap 1 and Hap 2 alleles that were identical to paternal alleles were classified as Type 1 and Type 2. Additional fetal sequences caused an imbalance of allele dosage compared to the maternal gDNA, as shown in the right part of the figure, and were used in deducing the fetal haplotype.

Haplotype phasing of maternal heterozygous SNPs was conducted based on family composition. We defined the maternal haplotype linked with the pathogenic variant as Hap1 and the other haplotype as Hap2. Then, according to fetal gender, we further classified Hap1 and Hap2 alleles into Type 1 and Type 2 (Figure 3). For a male fetus, the Type 1 and Type 2 alleles were the same as the Hap 1 and Hap 2 alleles, respectively. For a female fetus, if the paternal haplotype was available, then the Hap 1 and Hap 2 alleles identical to the paternal alleles were classified as Type 1 and Type 2. However, if the paternal haplotype was unavailable, then the method of classifying Type 1 and Type 2 alleles was the same as that of the male fetus. After haplotype phasing, quality control for SNP numbers was performed. When the number of Type 1 or Type 2 alleles was <10, this indicated consanguineous marriage. Haplotype-based NIPD was not suitable for such a family, and an invasive diagnosis was suggested.

To avoid the adverse influence of recombination events on RHDO, we predicted recombination events using the function “segmentByCBS” of the R package “PSCBS” (Olshen et al., 2011). Finally, based on allele frequency imbalance, we estimated the probability of inherited pathogenic haplotypes using the Bayesian method. Fetal allelic frequency was calculated separately using the following equation: $AF_{i}\;=AF_{cfDNA}^{i}-AF_{gDNA}^{i}$ , where $AF_{cfDNA}$ and $AF_{gDNA}$ are the allelic frequencies of maternal cfDNA and gDNA, respectively, and i is the SNP index. For each segment predicted, fetal Type 1 alleles were named $\;AF_{Type1}^{j}$ and fetal Type 2 alleles were designated $AF_{Type2}^{j}$ , where j is the index of segments. Then, we used the Bayes factor (BF) to predict maternally inherited haplotypes based on alleles positioned on segments where variants were located. This was the Bayes factor: $\frac{P\left(DC_{1}|H1\right)}{P\left(DC_{2}|H2\right)}.\;\;DC=AF_{type1}^{j}-AF_{type2}^{j}.$ Theoretically, the average dosage change (DC) between Type 1 and Type 2 alleles was *f*/2 or −*f*/2. H1  assumed that the fetus inherited Hap 1, and that the DC would be *f*/2. H2 presumed that the fetus inherited Hap 2, and that the DC would be −*f/2*. When BF≥10, we favored H1: the fetus was an affected male or a carrier female. However, when BF≤0.1, we favored H2 , and the fetus was classified as unaffected. A BF falling within the interval (0.1, 10) represented no call.

To evaluate the predicted comprehensive performance of fetal fraction, sequencing depth, and number of SNPs, we built an *in silico* model. The group of simulated allele dosage changes was randomly generated based on a binomial distribution by using quality control data from testing samples. The simulated process was repeated 1,000,000 times and the theoretical BF sensitivity, specificity, and no-call rate were then calculated based on the simulated data. If an expected sensitivity was <95%, another blood draw from the pregnant mother was performed 2 weeks later to obtain more reliable NIPD results.

### Validation of Fetal Genotypes

To validate accuracy, we performed invasive prenatal diagnosis for each family according to the results of NIPD by MLPA or Sanger sequencing, wherein chorionic villus sampling (CVS) for affected fetuses or amniocentesis for carriers and unaffected fetuses was performed.

## Results

### Confirmation of Germline Variants

After bioinformatics analysis, we successfully identified 13 large deletions (62.0%), 6 duplications (28.6%), and 2 point mutations (9.5%) at the *DMD* gene locus (Table 1). Specifically, 10 (76.9%) of the identified deletions were located at the region of exons 44–55, while five (83.3%) of the duplications were located in exons 8–12 (Supplementary Figures S2, S3). These two regions are hotspots for *DMD* gene variants. With targeted deep sequencing, the variants in the probands were validated and the mothers were defined as carriers. For families with deletions or duplications, the carrier mother had a different read depth when compared to the baseline read depth outside the deleted region, after normalization to reference datasets. Furthermore, all the identified variants were detected in both the proband and mother. The results indicated that it is an accurate method for DMD carrier screening.

### Measurement of Fetal Fraction and Gender

According to the autosomal loci homologous in both parents but with different genotypes, fetal fraction in maternal cfDNA was calculated, which ranged from 1.87 to 11.68% with a median of 6.21% (Table 1). However, because of low fetal fraction (0.86%) in family P5 after the first blood collection, which was lower than the quality control threshold (*f* ≥ 1%), we suggested another blood draw from the mother after 2 weeks to ensure detection accuracy.

**FIGURE 4:**
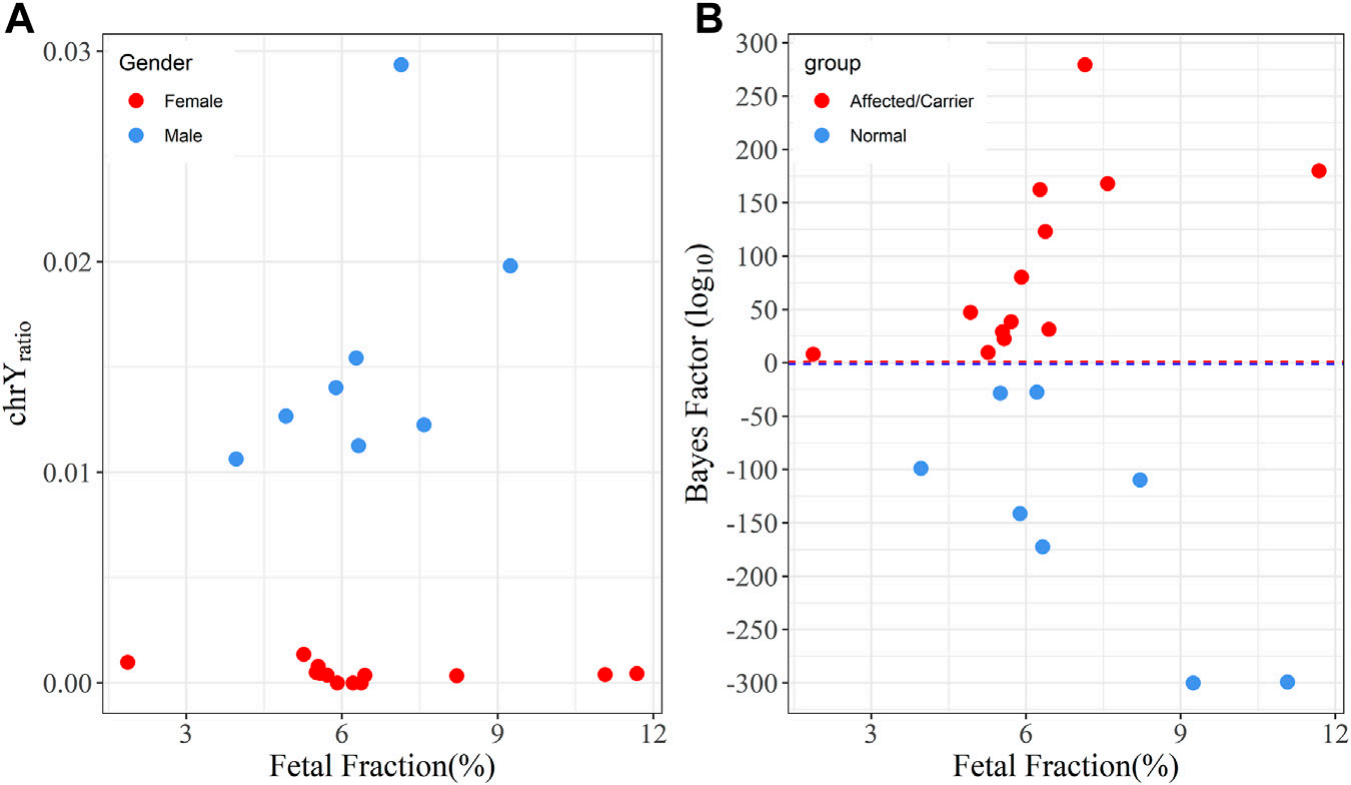
The relationship between fetal fraction, $\mathrm{chr}Y_{\mathrm{ratio}}$ and Bayes factors. **(A)** Relationship between fetal fraction and coverage ratio of chromosome. X-axis denotes fetal fraction. Y-axis denotes chrY_ratio_. Blue dots denote male fetuses and red dots indicate female fetuses. **(B)** Correlation of fetal fraction to Bayes factors. The X-axis denotes fetal fraction. The Y-axis indicates Bayes factors. Blue dots represent normal fetuses, and red dots depict affected or carrier fetuses.

Besides fetal fraction, gender determination is another critical factor for the NIPD of X-linked diseases. We used the ratio between the average depth of chromosome Y loci and 213 autosomal SNP loci (chrY_ratio) to predict fetal gender. In our dataset, we observed a significant difference in chrY_ratio between male and female fetuses, which were all <0.15% for female fetuses and >1% for male fetuses, indicating high accuracy in fetal gender determination (Figure 4A). Finally, we found 8 male fetuses and 13 female fetuses in all the enrolled families.

### NIPD of DMD

The average depth of gDNA ranged from 98× to 563×, the average on-target rate for gDNA was 65.38% (43.39–85.55%), and the average coverage (≥30×) of the target region was 91.86% (75.37–97.87%) (Supplementary Table S1). For each sample of maternal plasma cfDNA, the mean depth was 290× (range 165×–490×), and the average on-target rate was 62.71% (Supplementary Table S1). All samples met the depth quality control requirements and none of these required re-sequencing.

Based on family-based strategy and fetal gender, we further classified Hap 1 and Hap 2 into Type 1 and Type 2 as described in the methods section. The average number of Type 1 and Type 2 alleles was 310 (range: 97–687) and 303 (range: 108–687) (Table 1), respectively. The inherited allele was overrepresented in the maternal plasma. To evaluate the imbalance of fetal alleles, the Bayes factor (BF) was used to predict whether the fetal inherited maternal haplotype was pathogenic. The BF values (log_10_-transformation) showed a positive correlation with fetal fractions for fetuses inheriting Hap 1 (Pearson correlation coefficient r = 0.63, *p* = 0.021), while it presented a negative correlation for fetuses inheriting Hap 2 (r = -0.77, *p* = 0.024) (Figure 4B).

**FIGURE 5:**
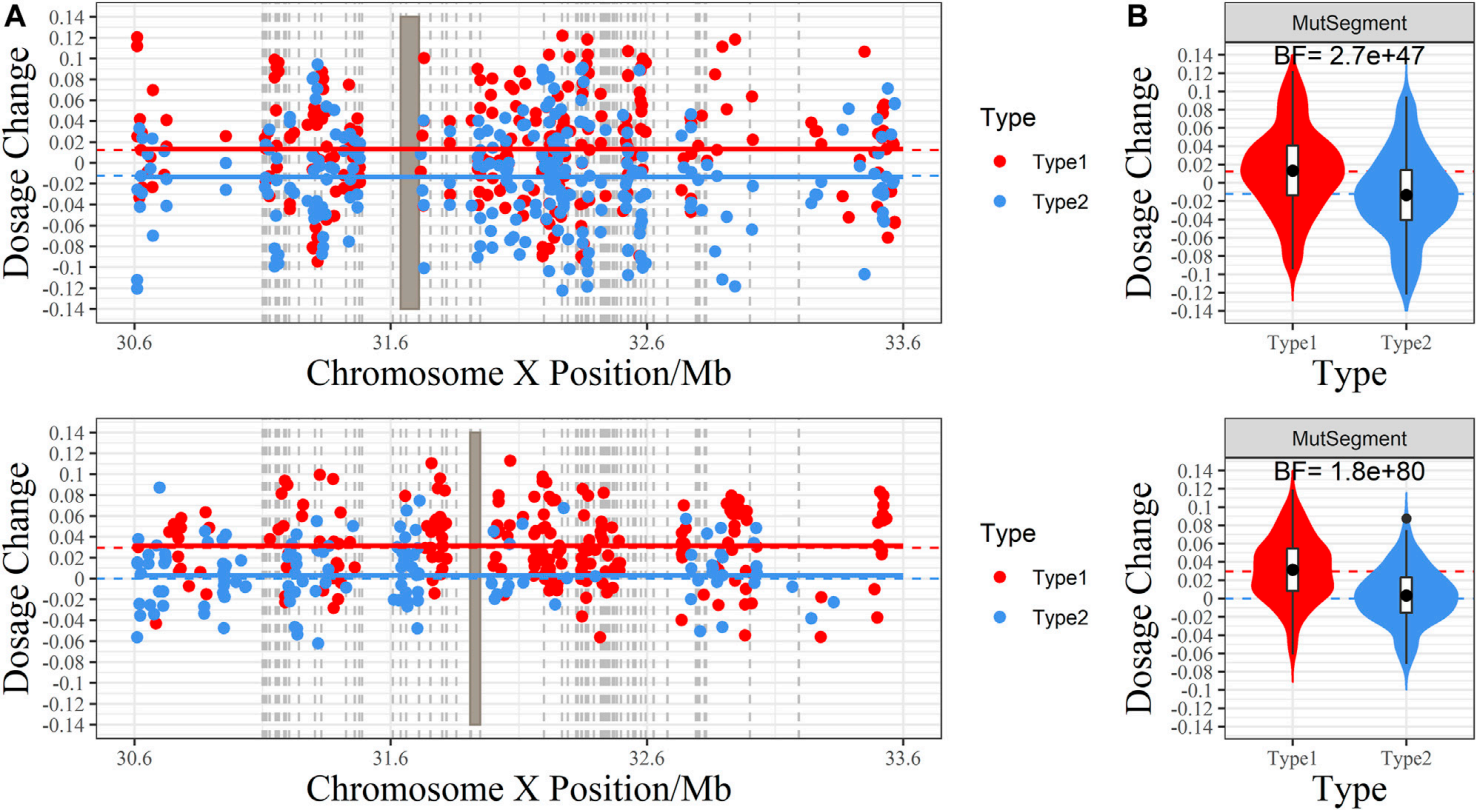
The RHDO results of families P1 and P2. **(A)** Scatter plot of the dosage change (DC) of each allele. The X-axis is the genomic coordinate, and the Y-axis represents DC. Red dots denote the DC of the Type 1 allele (over-represented when the fetus inherited maternal Hap1, which carries the pathogenic variant), whereas blue dots are the DC of the Type 2 allele (overrepresented if the fetus inherited maternal Hap 2, which carries the wild-type *DMD* gene). The red and blue dashed lines indicate the expected value of DC for Type 1 and Type 2 alleles under the assumption that the fetus inherits the maternal pathogenic and wild-type haplotype. The red and blue horizontal line is the center of DC returned by the CBS algorithm. When recombination occurs, both lines will cross at the switch site. Gray rectangles indicate the range of pathogenic deletion, and the gray vertical dashed line marks the position of *DMD* exons. **(B)** Violin plot of DC. The shape around each box demonstrates the distribution of DC. The red and blue dashed lines indicate the expected value of DC for Type 1 and Type 2 alleles under the assumption that the fetus inherits the maternal pathogenic and wild-type haplotypes. The Bayes factor is labeled at the top: BF ≥ 10 indicates a positive test result, favoring the assumption that the fetus inherits the maternal pathogenic variant, while a BF ≤ 0.1 reveals a negative test result, where the fetus inherits the wild-type haplotype.

BF showed significant accuracy in predicting fetal genotypes. For example, family P1 had a male fetus, whereas family P2 had a female fetus. The BFs of these two families were far more than 10 (2.7 ×  10^47^ for P1 and 1.8 ×  10^80^ for P2), indicating that the two fetuses inherited a maternal pathogenic haplotype (Figure 5). Taken together, combined with fetus gender, NIPD results revealed that four fetuses (P1, P6, P12, P16) were affected male patients, nine fetuses (P2, P8, P10, P11, P13, P14, P18, P19, P20) were female carriers (Supplementary Figure S5), and the others did not inherit maternal pathogenic haplotypes (Supplementary Figure S4). Positive and negative results were clearly differentiated (Figure 4B).

The recombination event largely influences the prediction accuracy of NIPD. In this study, recombination events downstream of the related variants were detected in two fetuses (P16, P18) by the CBS algorithm (Supplementary Figure S5). The recombination points were about 1.30 M (P16) and 0.59 M (P18) away from the related variants in these two families. The number of SNP linkages to variant positions was sufficient in generating precise NIPD results for the two families.

### Invasive Prenatal Diagnosis

To validate our NIPD results, we performed CVS for high-risk families and amniocentesis for low-risk families (Supplementary Figure S6). The results of all invasive diagnoses were similar to those generated by NIPD.

## Discussion

In our study, NIPD was successfully utilized to assess 21 fetuses at risk for DMD, with an average gestational age of 10^+3^ weeks. Fetal genotypes were detected by RHDO in maternal plasma DNA and subsequently confirmed by invasive prenatal diagnosis with 100% concordance. To our best knowledge, this is the largest dataset to date for the NIPD of DMD. Furthermore, it is also the first real-world dataset generated from clinical practice.

Earlier prenatal testing for DMD can provide families with more options to prepare, as well as more time for genetic therapy (Birnkrant et al., 2018; Iftikhar et al., 2021). The minimum gestational age in our study was 7^+3^ weeks (Family P11), which is 5 weeks earlier than CVS and 9 weeks earlier than amniocentesis. However, early gestational age often suggests a lower fetal fraction, which requires higher assay sensitivity (Lo et al., 2010). The minimum fetal fraction in our study was 1.87% (Family P10: 8^+0^ weeks), and the Bayes factor of that sample was still high enough (1.3  ×  10^8^) to support the *H*1. We defined the lower limit of the fetal fraction as 1%. In practice, only one family (1 in 21) was under that threshold at the first blood draw (Family P5: f = 0.86% at 7^+0^ weeks). The maternal blood of this family was recollected at 9^+0^ weeks, and the fetal fraction of the second sample ( f = 3.96%) then met the requirements. Based on the information mentioned above, we recommend the earliest blood collection time to be after 7^+0^ gestational weeks.

Instead of the straightforward detection of allelic mutations, we used linkage analysis for constructing the haplotype of the DMD region. By expanding the capture probe coverage from exonic regions to their adjacent intronic regions, combined with common SNPs in deep intronic regions, we successfully identified disease-causing variants in all proband and maternal gDNA samples in parallel with SNP genotyping, including exonic deletions and duplications. Of note, we even found the exact breakpoint in two families with an exonic deletion (data not shown). Our data indicated that target capture design could also be a powerful tool for the molecular diagnosis of DMD. For haplotype-based NIPD, the error risk caused by recombination within the *DMD* gene should be fully considered. Yoo et al. emphasized that recombination events within the *DMD* gene would greatly affect dosage imbalance analysis with false predictions, and deduced one recombination case in a duplication DMD family (Yoo et al., 2015). We utilized the circular binary segmentation (CBS) algorithm to predict the recombination event, which is widely used in detecting copy number variations. We found recombination in 2 of the 21 fetuses (P16 with a male fetus and P18 with a female fetus), and the observed frequency (11%) was concordant with the findings of previous reports (6–10%) (Abbs et al., 1990; Nobile et al., 1995; Shashi et al., 1996; Giliberto et al., 2011; Ling et al., 2020). Observed recombination sites of these two fetuses were both far away from the disease-causing region and did not interfere with inferring fetal genotypes. Subsequent invasive diagnosis confirmed the NIPD results.

A complete pedigree that includes parents and probands is necessary for prenatal diagnosis and genetic counseling (Cole et al., 1978; Posch et al., 2012; Slomp et al., 2018; Gilstrop Thompson et al., 2020). Nevertheless, in clinical practice, the situation of incomplete pedigrees occurs occasionally. In our dataset, two families (P12 and P18) lacked a proband, and six families (P1, P9, P13, P17, P19, and P21) lacked paternal samples. In families in which the proband was absent, we required normal offspring for haplotype phasing. Type 1 and Type 2 SNPs were swapped after haplotype phasing to predict the inheritance of the pathogenic haplotype. In families that lacked the paternal sample, the SNP classification between a male patient (P1) and a normal male fetus (P17, P21) was not influenced because paternal alleles did not act on haplotype dosage. However, if the fetus was a female, then dosage changes were not as expected for the portion of Type 1 and Type 2 SNPs in which paternal alleles differed from proband alleles, thereby decreasing the assay performance of distinguishing female carriers (P13, P19) and normal female fetuses (P9). However, we still reported the NIPD result in case of a definite Bayesian factor. We informed patients of the above information during pre-test genetic counseling.

Quality control is indispensable for the clinical application of NIPD. We performed quality control of each sequencing sample. The primary quality control metrics were fetal fraction, the number of informative SNPs, and sequencing depth. Besides setting up a lower limit for individual parameters, secondary quality control evaluated assay performance using an *in silico* model by considering all these parameters together. Supplementary Figure S7 demonstrates that the 3D surface comprises predicted assay performance (sensitivity and specificity), the number of informative SNPs, and sequencing depth at 1, 2, 4, and 8% fetal fraction. It was shown that even when the fetal fraction was as low as 1%, the predicted assay sensitivity could still be over 95% when the number of informative SNPs was larger than 200 and the sequencing depth was more than 500×. The *in silico* model provides clinicians a direct and simple tool for evaluating result reliability, therefore minimizing the chances of false positive NIPD results.

Additionally, testing costs and turnaround time are critical issues for widespread clinical applications. To optimize costs, we utilized a strategy for panel design with the selective enrichment of gene and spanning Single-Nucleotide Polymorphisms, which only targets essential genomic regions for NIPD. Using this approach, we narrowed down the panel size to only 288.612 kb, which was the minimum among previously reported panels dedicated to the NIPD of DMD (Xu et al., 2015; Yoo et al., 2015; Parks et al., 2016; Chen et al., 2019). Only an average of 5 M sequencing reads for maternal plasma DNA and 2 M sequencing reads for each family member’s gDNA were required, indicating that the required total number of reads per family is only 11 M. In addition, we mixed gDNA libraries from the same family together in the same proportion before target DNA capture to further reduce the cost for capture probes and hybridization reagents. As a result, experimental expense per family was controlled below $500, which is comparable to other routine genetic tests, such as family-based whole-exome sequencing (Fan et al., 2012; Kitzman et al., 2012; Rabinowitz et al., 2019). In terms of time effectiveness, blood samples were processed immediately after collection, followed by library construction. Probe hybridization was performed overnight. Post-capture libraries of NIPD shared one sequencing chip with other routine genetic tests such as noninvasive fetal aneuploidy testing (NIPT) or carrier screening to accelerate queuing time for next-generation sequencing. We developed a visual web application that integrates sample analysis, management, and report generation. This application liberates clinicians from complex bioinformatic analysis and saves manpower. By utilizing the strategies described above, the minimum turnaround time is shortened to 3 days. In most cases, test reports can be sent to patients within a week, which meets a typical genetic test requirement.

## Data Availability

The datasets for this article are not publicly available due to concerns regarding participant/patient anonymity. Requests to access the datasets should be directed to the corresponding authors.
