# Supplementary material for: Haplotype-Based Noninvasive Prenatal Diagnosis of 21 Families With Duchenne Muscular Dystrophy: Real-World Clinical Data in China
Source: Front Genet. 2021 Dec 14;12:791856. doi: 10.3389/fgene.2021.791856 (PMC8712857; doi:10.3389/fgene.2021.791856)
Supplement: Supplementary file 1 [file DataSheet1.docx]

**Haplotype-based noninvasive prenatal diagnosis of 21 families with Duchenne muscular dystrophy: Real-world clinical data in China**

Lingrong Kong^1,^ ^2†^; Shaojun Li^3†^; Zhenhua Zhao^2‡^; Jun Feng^3‡^; Guangquan Chen, ^1^; Lina Liu^2^; Weiqin Tang^3^; Suqing Li^3^; Feifei Li^3^; Xiujuan Han^3^; Di WU^3^*; Haichuan Zhang^3^*; Luming Sun^1^* and Xiangdong Kong^2^*

^1^ Department of Fetal Medicine & Prenatal Diagnosis Center,Shanghai First Maternity and Infant Hospital, School of Medicine, Tongji University, Shanghai, China

^2^Genetic and Prenatal Diagnosis Center, Department of Obstetrics and Gynecology,

The First Affiliated Hospital of Zhengzhou University, Zhengzhou, China.

^3^Celula (China) Medical Technology Co., Ltd., Chengdu, China.

**^*^Correspondence**:

Xiangdong, Kong ([kongxd@zzu.edu.cn](mailto:kongxd@zzu.edu.cn))

Luming, Sun (Luming_sun@163.com)

Haichuan Zhang (hzhang@neoseqltd.com)

Di Wu ([james.wu@celula-china.com](mailto:james.wu@celula-china.com))

^†^These authors share first authorship.

^‡^These authors share senior authorship.


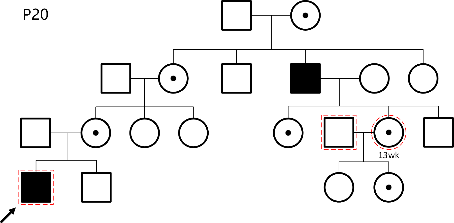

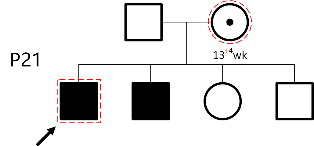

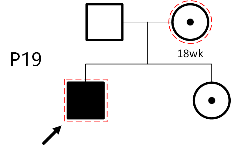


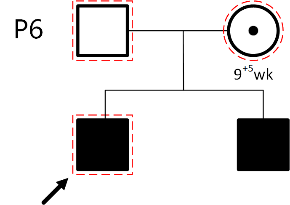

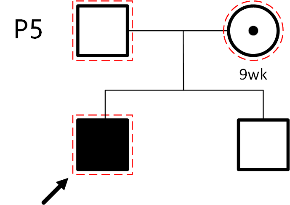

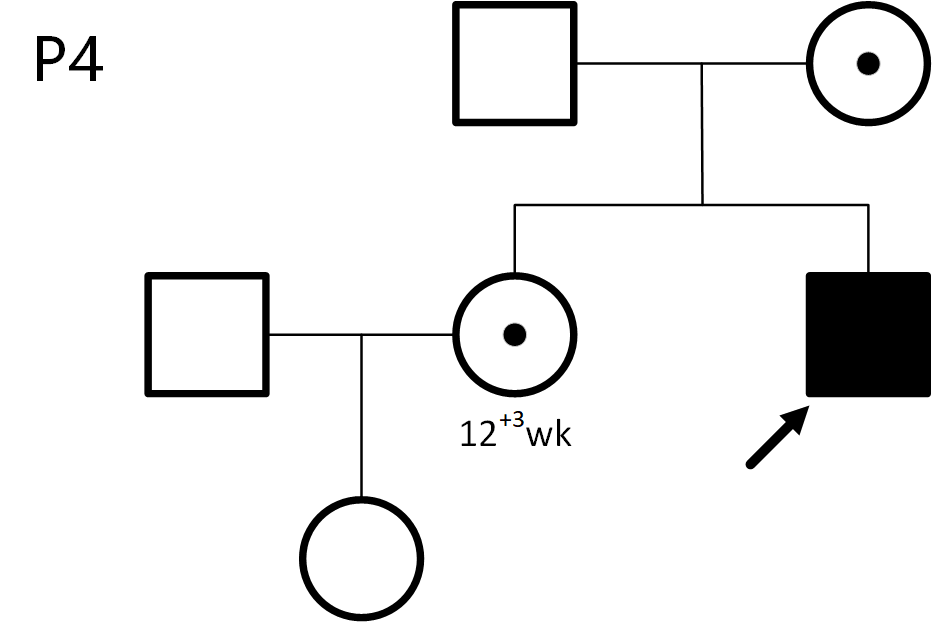

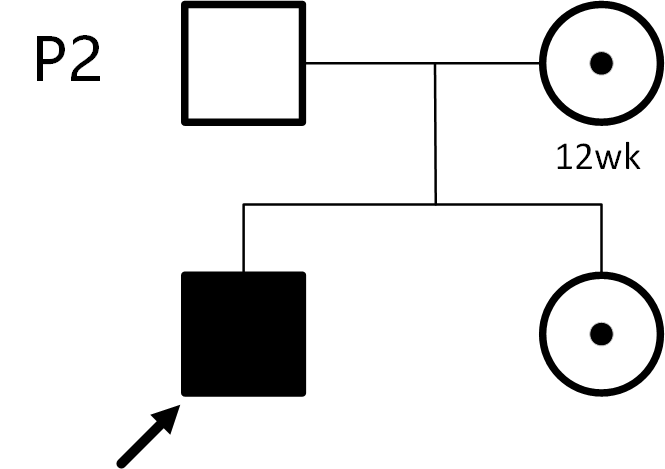

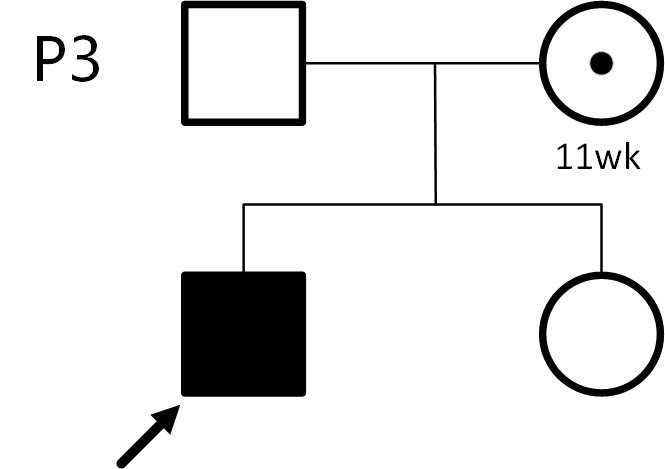


P1: Exon 53_55 deletion

P2: Exon 46_48 deletion

P3:Exon 12_13 duplication

P4:c.3055C>T (p.Q1019*)

P5: Exon45_50deletion

P6: c.3786+2T>A

P7:Exon10_11duplication

P8: Exon3_4deletion

P9: Exon8_26deletion

P10: Exon45_51deletion

P11: Exon10_13deletion

P12: Exon48_50deletion

P13: Exon8_9duplication

P14: Exon50deletion

P15: Exon45_55deletion

P16: Exon46_51deletion

P17:Exon3_25duplication

P18: Exon48_52deletion

P19: Exon8_9duplication

P20: Exon45_47deletion

P21: Exon8_9duplication


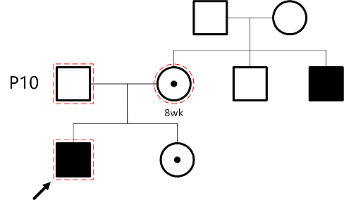

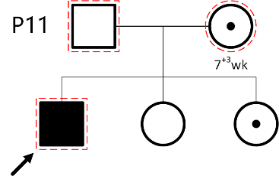

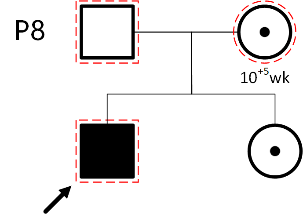

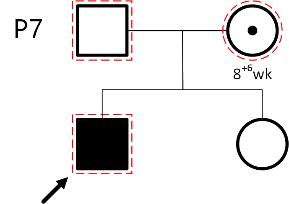

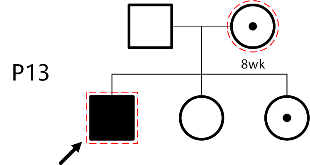

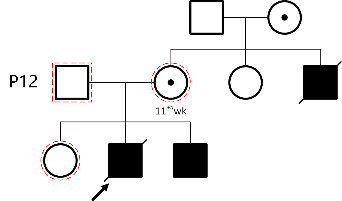

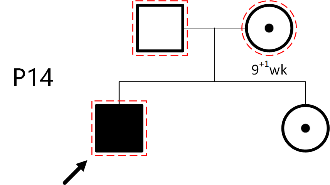

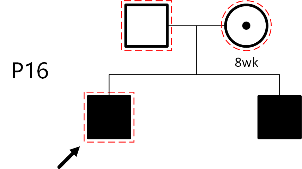

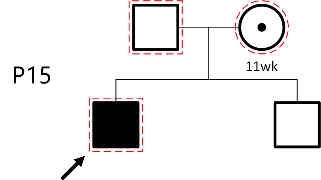

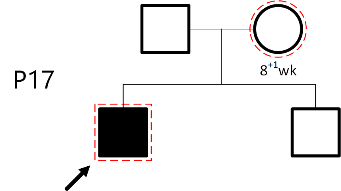

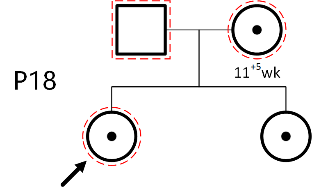

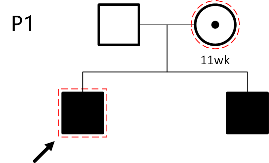

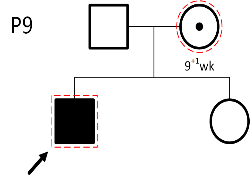


**Figure S1**. **Pedigree of families**: The inherited variants were identified in the *DMD* gene for the eleven recruited families. The male probands are indicated by arrows. The week of gestation (wk) of the mother (pregnant) for each family is shown in the figure. The mothers in these families were heterozygote with genotypes of one mutant allele and one wild-type allele. P4 proband was the brother of the pregnant woman. The red dotted frame represents family members with the venous puncture to obtain the whole blood.


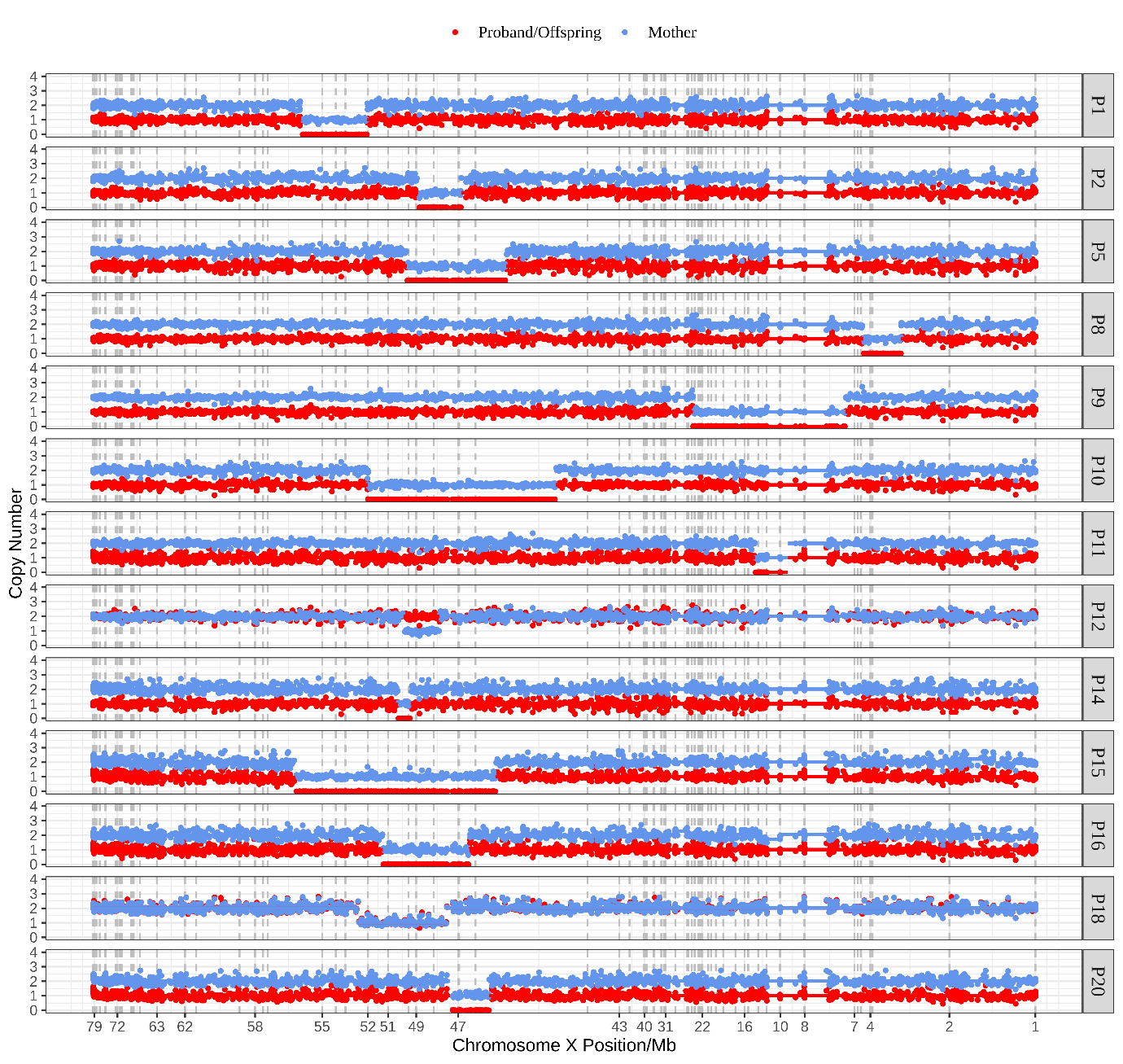


**Figure S2 The consistence of variant of *DMD* gene deletion between mother and proband/offspring**. The X-axis represents chromosome X position labeled by exon number of *DMD* gene. The Y-axis represents copy number. The blue (mother) and red (proband/offspring) dots represent a copy number values of a specific sliding window. The red and blue lines represent the copy number identified by the CBS algorithm.The results showed that offsprings of eleven families (P1, P2, P5, P8, P9, P10, P11,P14, P15, P16, P20) were DMD patients and offspring of one families (P18) were *DMD* gene deletion carrier (EX48-52), where the variants inherited from their mother. For family P12, the offspring was normal and the mother was a *DMD* gene deletion carrier.


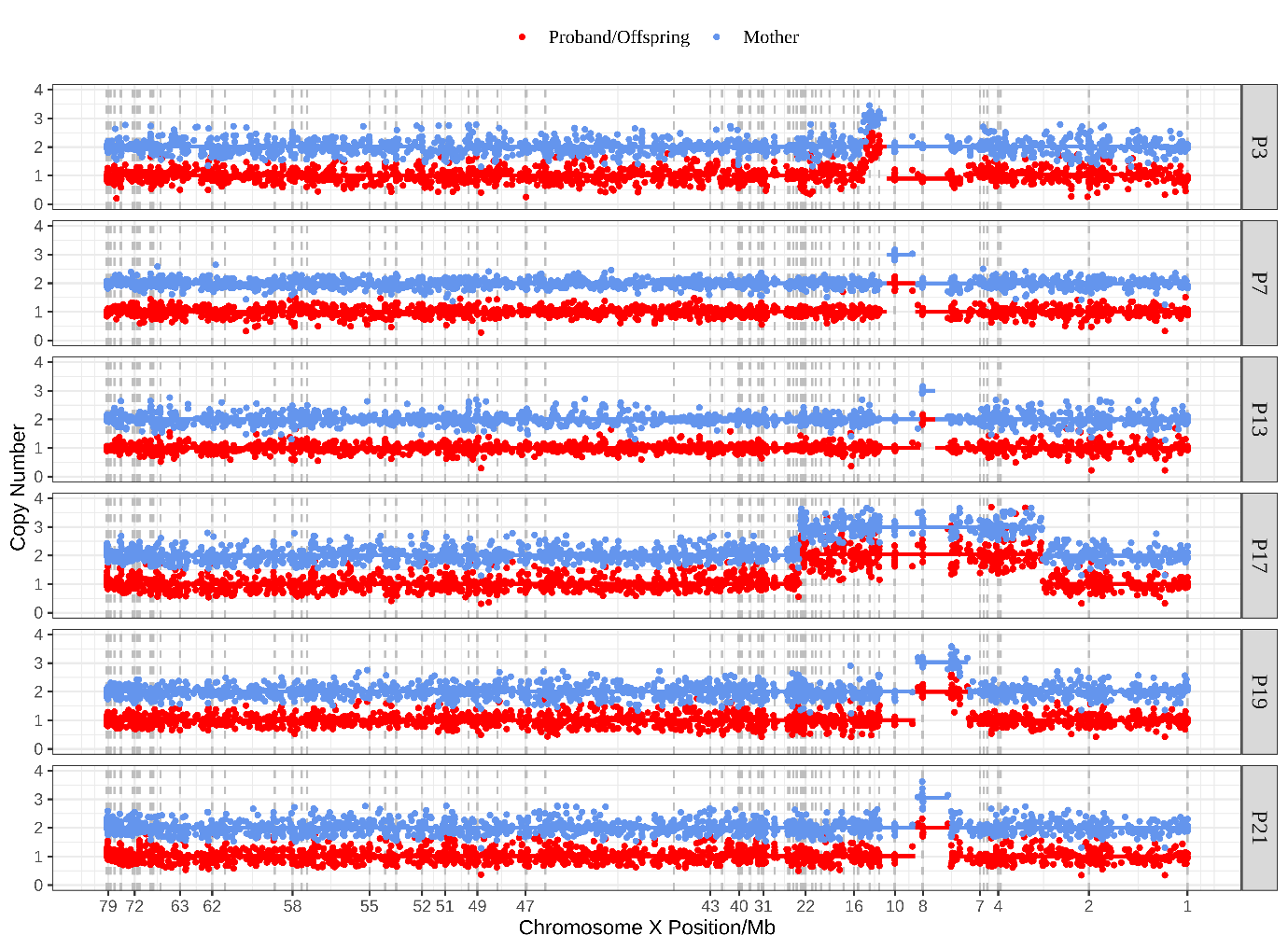


**Figure S3 The consistence of variant of *DMD* gene duplication between mother and proband/offspring**. The X-axis represents chromosome X position labeled by exon number of *DMD* gene. The Y-axis represents copy number. The blue (mother) and red (proband/offspring) dots represent a copy number values of a specific sliding window. The red and blue lines represent the copy number identified by the CBS algorithm.The results showed that all the offspring were DMD patients, where the variants inherited from their mother.


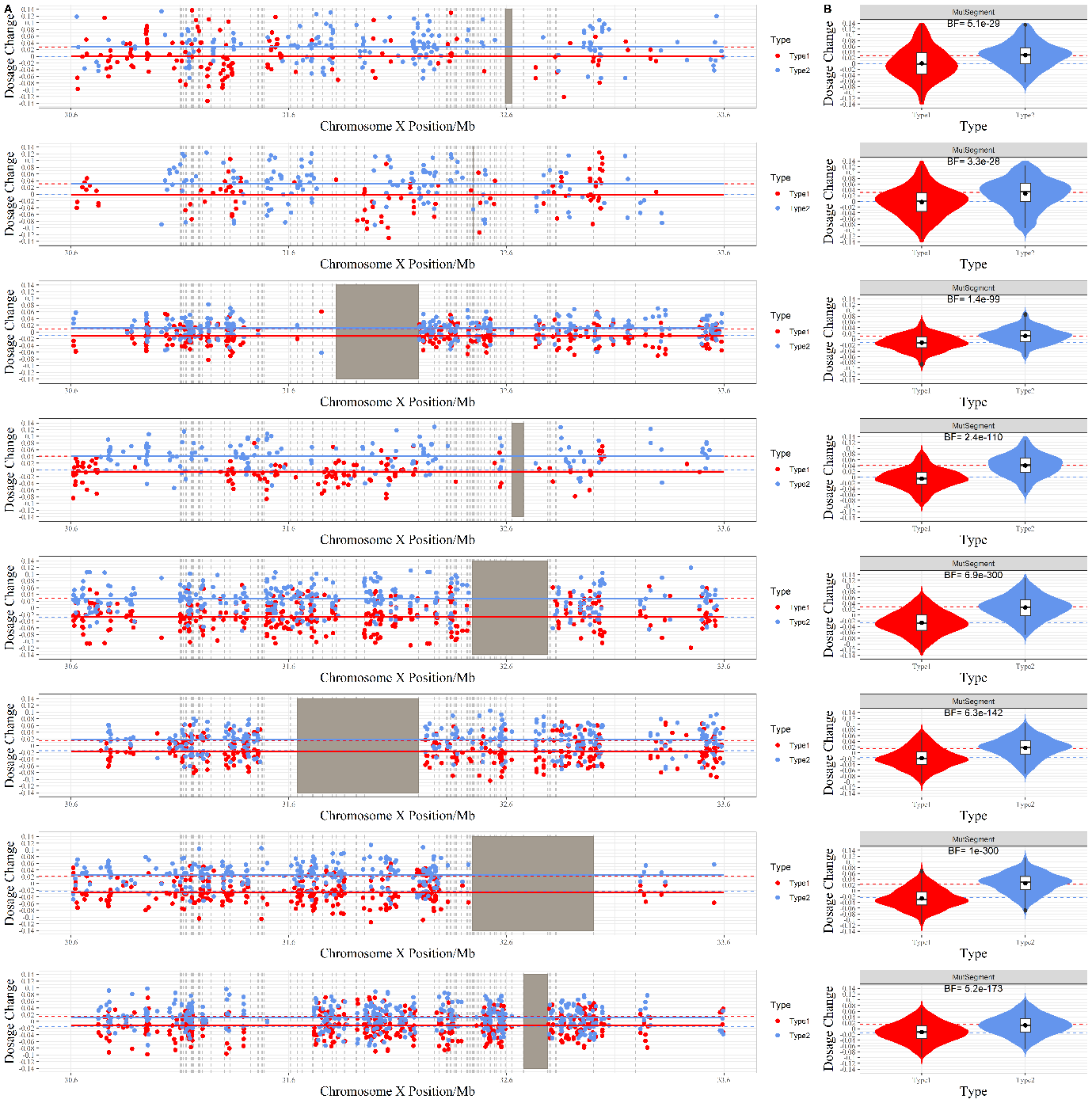


**Figure S4. The RHDO results of family with normal fetuses.** A) Scatter plot of the dosage change (DC) of each allele for each family. The families from up to bottom were P3, P4, P5, P7, P9, P15, P17, P21. The X-axis is genomic coordinate, and the Y-axis represents DC. Red dots denote the DC of Type 1 allele (over-represented if the fetus inherited maternal Hap1, which carries the pathogenic variant), while blue dots are the DC of Type 2 allele (over-represented if the fetus inherited maternal Hap 2, which carries wild-type *DMD* gene). The red and blue dashed lines indicate the expected value of DC for Type1 and Type2 alleles under the assumption that the fetus inherits maternal pathogenic and wild-type haplotype.The red and blue horizontal line is the center of DC returned by the CBS algorithm. When recombination event presents, both lines will cross-over at the switch-site. Gray rectangles indicate the range of pathogenic deletion, and the gray vertical dashed line marks the position of *DMD* exons. B) Violin plot of DC. The shape around each box demonstrates the distribution of DC. The red and blue dashed lines indicate the expected value of DC for Type1 and Type2 alleles under the assumption that the fetus inherits maternal pathogenic and wild-type haplotype. The Bayes factor is labeled on the top region, BF≥10 indicates a positive test result, favoring the assumption that the fetus inherits maternal pathogenic variant, while BF≤0.1 reveals a negative test result, where the fetus inherits the wild-type haplotype.


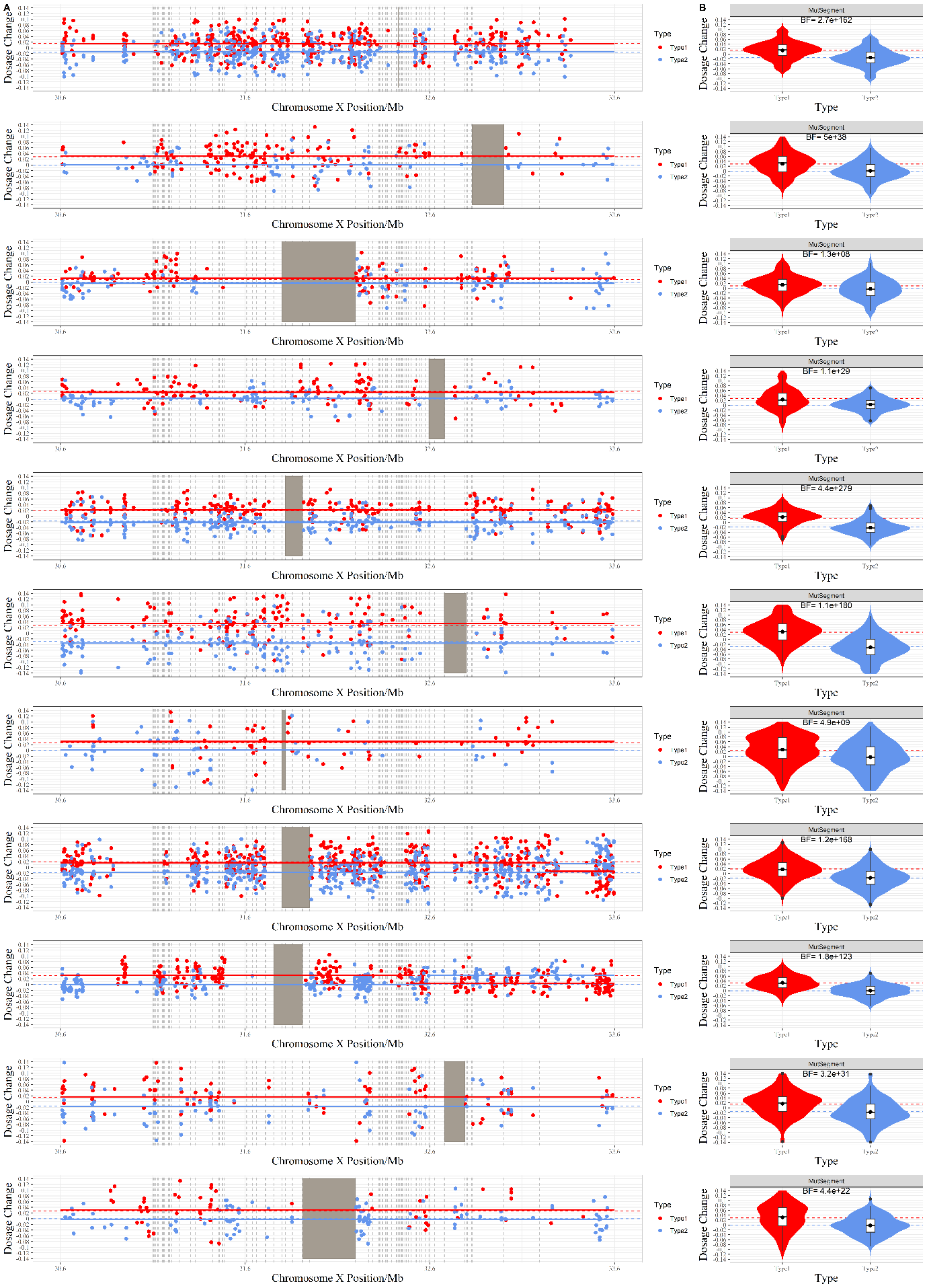


**Figure S5. The RHDO results of family with carrier/affected fetuses.** A) Scatter plot of the dosage change (DC) of each allele for each family. The families from up to bottom were P6, P8, P10, P11, P12, P13, P14, P16, P18, P19, P20.The X-axis is genomic coordinate, and the Y-axis represents DC. Red dots denote the DC of Type 1 allele (over-represented if the fetus inherited maternal Hap1, which carries the pathogenic variant), while blue dots are the DC of Type 2 allele (over-represented if the fetus inherited maternal Hap 2, which carries wild-type *DMD* gene). The red and blue dashed lines indicate the expected value of DC for Type1 and Type2 alleles under the assumption that the fetus inherits maternal pathogenic and wild-type haplotype.The red and blue horizontal line is the center of DC returned by the CBS algorithm. When recombination event presents, both lines will cross-over at the switch-site. Gray rectangles indicate the range of pathogenic deletion, and the gray vertical dashed line marks the position of *DMD* exons. B) Violin plot of DC. The shape around each box demonstrates the distribution of DC. The red and blue dashed lines indicate the expected value of DC for Type1 and Type2 alleles under the assumption that the fetus inherits maternal pathogenic and wild-type haplotype. The Bayes factor is labeled on the top region, BF≥10 indicates a positive test result, favoring the assumption that the fetus inherits maternal pathogenic variant, while BF≤0.1 reveals a negative test result, where the fetus inherits the wild-type haplotype.


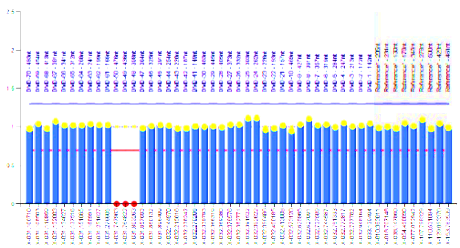


**P12**


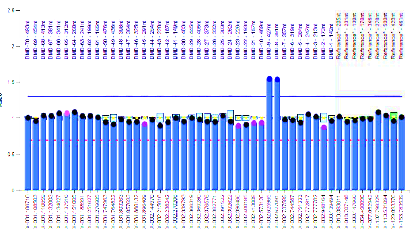


**P13**


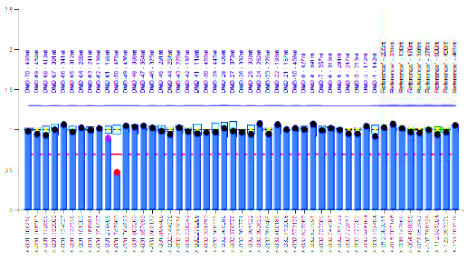


**P14**


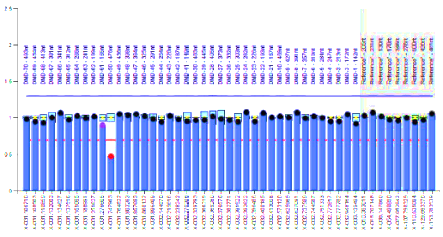


**P15**


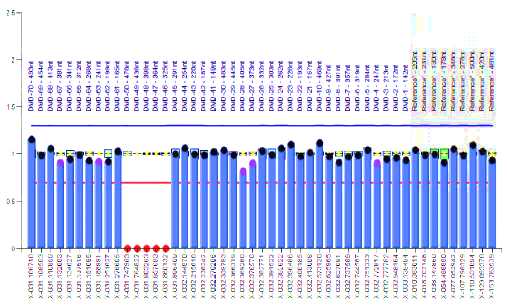


**P16**


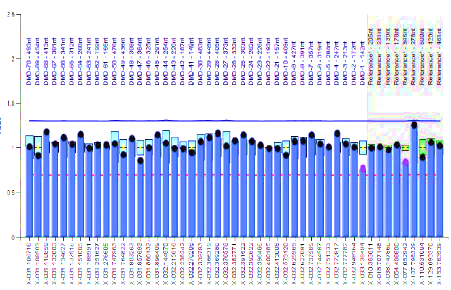


**P17**


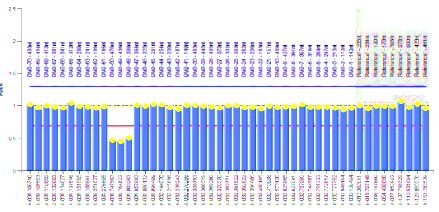


**P18**


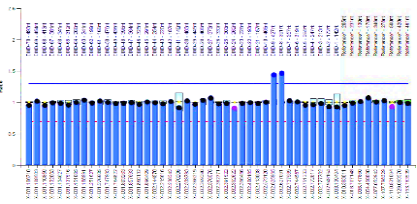

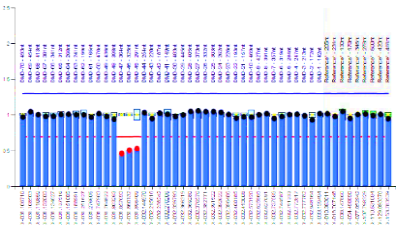

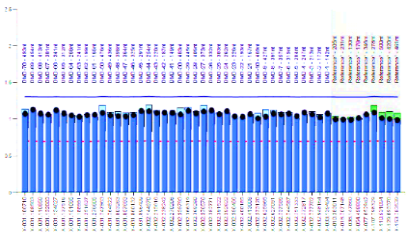


**P19**

**P20**

**P21**

P1


P2

P3

**P1**G

**P2**G

**P11**G

**P10**G

**P9**G

**P8**

**P7**G

**P5**G

**P3**G

**Figure S6. The invasive procedure results of recruited families:** P4 and P6 have single nucleotide variations and were verified by Sanger Sequencing, while other families were confirmed by MLPA.

**
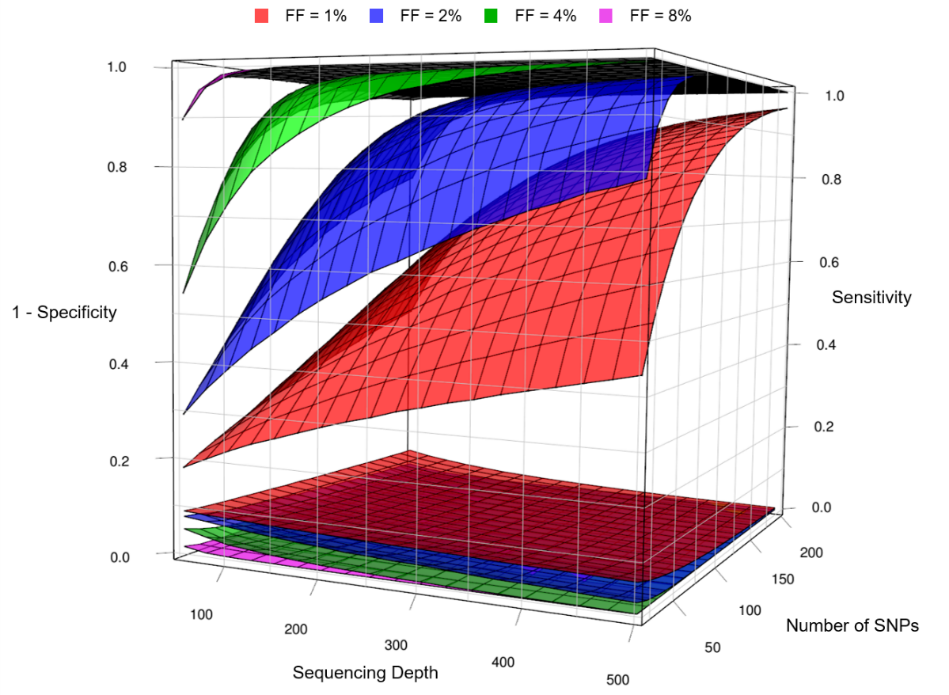
**

**Figure S7. Simulation of assay performance.** Grid-search of sequencing depth (50-500x at a 50 step size) and the number of SNPs (20-200 at a 20 step size) were conducted for 1%, 2%, 4%, 8% fetal fraction separately. At each condition, sampling base on binomial distribution was repeated 500,000 times. The constructed simulation model was plotted into a 3D space. The X-axis represents sequencing depth; the Y-axis represents the number of SNPs; colors denote different fetal fractions. Curved surfaces at the upper part of the figure are the simulated sensitivities (refer to the Z-axis at the right-rear edge), while curved surfaces at the lower part are the simulated 1 – specificity values (refer to the Z-axis at the left-front edge).

**Table S1** The family information and high-throughput datasets of the 21 DMD families.

| Family | Sample Name | Relationship^1^ | Gender | Sample Type | Variants | Genotype | TotalReads | Average depth | on-target rate% | Coverage 30X |
| --- | --- | --- | --- | --- | --- | --- | --- | --- | --- | --- |
| P1 | P1-2 | Proband | M | GDNA | EX53_55del | hemi | 1400847 | 291 | 80.29 | 93.95% |
|  | P1-3 | Mother | F | GDNA | EX53_55del | het | 1048240 | 146 | 51.05 | 91.10% |
|  | P1-1 | Mother | F | CFDNA | - | - | 3221968 | 334 | 47.44 | 83.51% |
| P2 | P2-2 | Proband | M | GDNA | EX46_48del | hemi | 2305092 | 420 | 78.59 | 94.75% |
|  | P2-3 | Mother | F | GDNA | EX46_48del | het | 1484297 | 257 | 60.77 | 94.37% |
|  | P2-4 | Father | M | GDNA | - | - | 1690564 | 328 | 78.75 | 95.52% |
|  | P2-1 | Mother | F | CFDNA | - | - | 3055626 | 339 | 69.04 | 86.48% |
| P3 | P3-1 | Proband | M | GDNA | EX12_13dup | hemi | 1579265 | 236 | 67.89 | 91.30% |
|  | P3-2 | Mother | F | GDNA | EX12_13dup | het | 1434867 | 290 | 83.04 | 92.10% |
|  | P3-3 | Father | M | GDNA | - | - | 1309095 | 209 | 70.55 | 90.55% |
|  | P3-4 | Mother | F | CFDNA | - | - | 3443023 | 360 | 61.96 | 82.66% |
| P4 | P4-2 | Proband | M | GDNA | c.3055C>T | hemi | 1200016 | 218 | 68.63 | 86.93% |
|  | P4-3 | Mother | F | GDNA | c.3055C>T | het | 1528663 | 325 | 77.51 | 91.98% |
|  | P4-4 | Father | M | GDNA | - | - | 1370659 | 272 | 72.09 | 92.95% |
|  | P4-1 | Mother | F | CFDNA | - | - | 4802950 | 490 | 66.17 | 79.26% |
| P5 | P5-1 | Mother | F | GDNA | EX45_50del | het | 1391599 | 261 | 65.50 | 94.32% |
|  | P5-3 | Proband | M | GDNA | EX45_50del | hemi | 1025466 | 180 | 54.56 | 81.23% |
|  | P5-4 | Father | M | GDNA | - | - | 774986 | 138 | 55.80 | 77.72% |
|  | P5-2 | Mother | F | CFDNA | - | - | 4361138 | 487 | 55.42 | 92.87% |
| P6 | P6-1 | Proband | M | GDNA | c.3786T+2>A | hemi | 1026385 | 205 | 74.58 | 93.47% |
|  | P6-2 | Mother | F | GDNA | c.3786T+2>A | het | 1398882 | 328 | 85.22 | 91.04% |
|  | P6-3 | Father | M | GDNA | - | - | 898033 | 181 | 76.21 | 92.55% |
|  | P6-4 | Mother | F | CFDNA | - | - | 3807118 | 443 | 67.08 | 88.52% |
| P7 | P7-1 | Proband | M | GDNA | EX10_11dup | hemi | 997831 | 149 | 56.40 | 89.09% |
|  | P7-2 | Mother | F | GDNA | EX10_11dup | het | 1327575 | 229 | 64.78 | 92.51% |
|  | P7-3 | Father | M | GDNA | - | - | 905860 | 138 | 57.16 | 88.40% |
|  | P7-4 | Mother | F | CFDNA | - | - | 3241800 | 349 | 75.00 | 79.00% |
| P8 | P8-1 | Proband | M | GDNA | EX3_4del | hemi | 1218135 | 209 | 69.06 | 92.65% |
|  | P8-2 | Mother | F | GDNA | EX3_4del | het | 1665695 | 344 | 81.65 | 95.10% |
|  | P8-3 | Father | M | GDNA | - | - | 1142808 | 209 | 70.20 | 93.84% |
|  | P8-4 | Mother | F | CFDNA | - | - | 2939307 | 298 | 80.00 | 79.78% |
| P9 | P9-1 | Proband | M | GDNA | EX8_26del | hemi | 1932048 | 303 | 73.55 | 91.36% |
|  | P9-3 | Mother | F | GDNA | EX8_26del | het | 1606580 | 264 | 78.38 | 96.31% |
|  | P9-2 | Mother | F | CFDNA | - | - | 3180057 | 293 | 79.73 | 83.94% |
| P10 | P10-1 | Proband | M | GDNA | EX45_51del | hemi | 1342948 | 204 | 50.68 | 82.88% |
|  | P10-2 | Father | M | GDNA | - | - | 886492 | 138 | 50.72 | 79.74% |
|  | P10-3 | Mother | F | GDNA | EX45_51del | het | 906837 | 129 | 45.07 | 87.73% |
|  | P10-4 | Mother | F | CFDNA | - | - | 1974090 | 178 | 43.38 | 81.75% |
| Family | Sample Name | Relationship^1^ | Gender | Sample Type | Variants | Genotype | TotalReads | Average depth | on-target rate% | Coverage 30X |
| P11 | P11-1 | Proband | M | GDNA | EX10_13del | hemi | 1039340 | 186 | 63.75 | 93.38% |
|  | P11-2 | Mother | F | GDNA | EX10_13del | het | 2112511 | 387 | 66.87 | 95.57% |
|  | P11-3 | Father | M | GDNA | - | - | 1047714 | 184 | 62.26 | 93.99% |
|  | P11-4 | Mother | F | CFDNA | - | - | 2281892 | 222 | 69.33 | 77.90% |
| P12 | P12-1 | Offspring | F | GDNA | - | - | 1109254 | 188 | 52.62 | 86.23% |
|  | P12-2 | Mother | F | GDNA | EX48_50del | het | 1960350 | 320 | 65.42 | 96.16% |
|  | P12-3 | Father | M | GDNA | - | - | 641294 | 98 | 47.95 | 75.37% |
|  | P12-4 | Mother | F | CFDNA | - | - | 1905634 | 165 | 48.92 | 93.19% |
| P13 | P13-1 | Proband | M | GDNA | EX8_9dup | hemi | 1135552 | 250 | 82.89 | 87.81% |
|  | P13-2 | Mother | F | GDNA | EX8_9dup | het | 1444893 | 192 | 53.76 | 86.54% |
|  | P13-3 | Mother | F | CFDNA | - | - | 2632065 | 226 | 57.14 | 78.56% |
| P14 | P14-1 | Proband | M | GDNA | EX50del | hemi | 1547134 | 192 | 57.25 | 84.39% |
|  | P14-2 | Mother | F | GDNA | EX50del | het | 1666031 | 246 | 58.66 | 96.76% |
|  | P14-3 | Father | M | GDNA | - | - | 1576602 | 180 | 54.61 | 82.97% |
|  | P14-4 | Mother | F | CFDNA | - | - | 2215120 | 228 | 52.44 | 76.62% |
| P15 | P15-1 | Proband | M | GDNA | EX45_55del | hemi | 1193337 | 184 | 61.82 | 90.70% |
|  | P15-2 | Mother | F | GDNA | EX45_55del | het | 2432677 | 427 | 72.77 | 97.12% |
|  | P15-3 | Father | M | GDNA | - | - | 933846 | 151 | 65.74 | 96.05% |
|  | P15-4 | Mother | F | CFDNA | - | - | 2887629 | 310 | 65.74 | 87.48% |
| P16 | P16-1 | Proband | M | GDNA | EX46_51del | hemi | 1902982 | 311 | 67.00 | 94.73% |
|  | P16-2 | Mother | F | GDNA | EX46_51del | het | 3509290 | 563 | 73.71 | 97.21% |
|  | P16-3 | Father | M | GDNA | - | - | 2275875 | 369 | 65.83 | 97.87% |
|  | P16-4 | Mother | F | CFDNA | - | - | 2717045 | 173 | 51.92 | 95.89% |
| P17 | P17-1 | Proband | M | GDNA | EX3_25dup | hemi | 1821463 | 265 | 54.60 | 97.27% |
|  | P17-2 | Mother | F | GDNA | EX3_25dup | het | 1969912 | 321 | 67.43 | 97.24% |
|  | P17-3 | Mother | F | CFDNA | - | - | 2301613 | 238 | 77.64 | 85.09% |
| P18 | P18-1 | Offspring | F | GDNA | EX48_52del | het | 2450120 | 403 | 65.75 | 97.08% |
|  | P18-2 | Mother | F | GDNA | EX48_52del | het | 2721699 | 450 | 65.87 | 97.17% |
|  | P18-3 | Father | M | GDNA | - | - | 1936888 | 283 | 57.44 | 97.58% |
|  | P18-4 | Mother | F | CFDNA | - | - | 3112934 | 327 | 59.93 | 95.76% |
| P19 | P19-1 | Proband | M | GDNA | EX8_9dup | hemi | 1473789 | 230 | 72.12 | 93.39% |
|  | P19-2 | Mother | F | GDNA | EX8_9dup | het | 2216352 | 390 | 83.55 | 95.20% |
|  | P19-3 | Mother | F | CFDNA | - | - | 2249718 | 188 | 77.58 | 79.72% |
| P20 | P20-1 | Proband | M | GDNA | EX45_47del | hemi | 1173368 | 133 | 43.59 | 91.02% |
|  | P20-2 | Mother | F | GDNA | EX45_47del | het | 1668966 | 206 | 45.10 | 95.61% |
|  | P20-3 | Father | M | GDNA | - | - | 1358639 | 151 | 43.39 | 92.31% |
|  | P20-4 | Mother | F | CFDNA | - | - | 2510715 | 233 | 48.32 | 82.31% |
| P21 | P21-1 | Proband | M | GDNA | EX8_9dup | hemi | 1546358 | 326 | 70.91 | 96.58% |
|  | P21-2 | Mother | F | GDNA | EX8_9dup | het | 1557924 | 377 | 85.55 | 97.09% |
|  | P21-3 | Mother | F | CFDNA | - | - | 2289206 | 206 | 62.79 | 93.92% |

^1^: All the proband are offspring except for family P4, who is the brother of the pregnant.

| **Table S2** Comparison of the current NIPD test for DMD with previously reported tests. | | | | | | | |  |
| --- | --- | --- | --- | --- | --- | --- | --- | --- |
| **PMID** | **Sample number** | **RHDO algorithm** | **Breakpoint** | **Probe design** | **Probe Size** | **cost** |  |  |
|  |  |  |  |  |  |  |  | |
| 25654318(Xu et al., 2015) | 8 | HMM | NO | Exonic regions & chromosome 22 | 1.66M | Not Given |  | |
|  |  |  |  |  |  |  |  | |
| 25847990(Yoo et al., 2015) | 4 | Student’s paired t-test &Wilcoxon signed-rank test | Pindel | all exons & introns | Not Given | Not Given |  | |
|  |  |  |  |  |  |  |  | |
| 26824862(Parks et al., 2016) | 2 | SPRT | NO | Long region containing the *DMD* gene | 2.4Mb | £640 |  | |
|  |  |  |  |  |  |  |  | |
| 31207536(Chen et al., 2019) | 17 | HMM | NO | Exonic regions & chromosome Y | 657.29Kb | Not Given |  | |
|  |  |  |  |  |  |  |  | |
| Current method | 21 | BF | STARS | enrichment of DMD gene exons and spanning SNP | 288.612Kb | $464 |  | |

**REFERENCES**

Chen, M., Chen, C., Li, Y., Yuan, Y., Lai, Z., Guo, F., et al. (2019). Haplotype-Based noninvasive prenatal diagnosis for duchenne muscular dystrophy: A pilot study in South China. *Eur J Obstet Gynecol Reprod Biol* 240**,** 15-22. doi: 10.1016/j.ejogrb.2019.05.005.

Parks, M., Court, S., Cleary, S., Clokie, S., Hewitt, J., Williams, D., et al. (2016). Non-invasive prenatal diagnosis of Duchenne and Becker muscular dystrophies by relative haplotype dosage. *Prenat Diagn* 36(4)**,** 312-320. doi: 10.1002/pd.4781.

Xu, Y., Li, X., Ge, H.J., Xiao, B., Zhang, Y.Y., Ying, X.M., et al. (2015). Haplotype-based approach for noninvasive prenatal tests of Duchenne muscular dystrophy using cell-free fetal DNA in maternal plasma. *Genet Med* 17(11)**,** 889-896. doi: 10.1038/gim.2014.207.

Yoo, S.K., Lim, B.C., Byeun, J., Hwang, H., Kim, K.J., Hwang, Y.S., et al. (2015). Noninvasive prenatal diagnosis of duchenne muscular dystrophy: comprehensive genetic diagnosis in carrier, proband, and fetus. *Clin Chem* 61(6)**,** 829-837. doi: 10.1373/clinchem.2014.236380.
